# Breastfeeding is not a risk factor for clinical severity in Autism spectrum disorder in children from the ELENA cohort

**DOI:** 10.1038/s41598-022-27040-x

**Published:** 2023-01-16

**Authors:** Marianne Peries, Fanny Duhr, Marie-Christine Picot, Barbara Heude, Jonathan Y. Bernard, Amaria Baghdadli

**Affiliations:** 1 Centre de Ressource Autisme Languedoc-Roussillon, CHU Montpellier, Montpellier, France; 2 Centre d’Excellence sur l’Autisme et les Troubles Neuro-développementaux (CeAnd), CHU Montpellier, Montpellier, France; 3 Université Paris-Saclay, UVSQ, Inserm, CESP, Team DevPsy, 94807 Villejuif, France; 4 Clinical Research and Epidemiology Unit, Department of Medical Information, University Hospital, CHU Montpellier, Montpellier, France; 5 Université Paris Cité, Inserm, INRAE, Centre de Recherche en Épidémiologie et Statistiques, F-7005 Paris, France; 6 Singapore Institute for Clinical Sciences (SICS), Agency for Science, Technology and Research (A*STAR), Singapore, Singapore; 7 Faculté de Médecine, Université de Montpellier, Montpellier, France

## Abstract

Autism spectrum disorder (ASD) is a neurodevelopmental disorder that results from a complex interaction between genes and environment. Breastfeeding (BF) is thought to promote healthy cognitive development, and a body of research has suggested that it may also protect against ASD. Our objectives were to identify the relationship between the initiation and duration of BF and the severity of clinical presentation in ASD. Data were collected from 243 children with a confirmed diagnosis of ASD followed in the ELENA cohort. Clinical severity was measured according to multiple dimensions using standardised tools. The frequency of the initiation of BF was comparable to that of the general population and the rate of children still being breastfed at six months of age was higher. Our results did not indicate a contribution of initiation or duration of BF to the prevention of clinical severity of ASD. We discuss our results in the light of possible methodological limitations of previous reports of an association between BF and ASD.

**Clinical Trial Registration**: NCT02625116.

## Introduction

Autism spectrum disorder (ASD) is a persistent neurodevelopmental disability. It is clinically evident from early childhood and related to brain development^1^. The characteristics of clinical presentation can be very heterogeneous in terms of both severity and outcome^2^. The risk is believed to be multifactorial, related to an interplay of genetic and environmental factors during critical perinatal windows. A growing number of studies are focusing on the nutritional status of children with ASD^3–5^.

Maternal malnutrition during pregnancy has been found to be associated with an increased risk of adverse neurodevelopmental outcomes in offspring^6–8^. There is also evidence of an association between breastfeeding (BF) and better cognitive outcome^9–11^, particularly when BF is exclusive^10–14^. This positive impact of BF could be due to the nutritional or hormonal content of breast-milk or to the social contact between mother and child during the act of nursing^15^. However, conflicting literature exists regarding the consistency of this positive impact attributed to BF after adjustment for important covariates such as parental education and socio-economic status. This suggests caution in the interpretation of these results^16^.

Studies on the association between BF and ASD show conflicting results. Two recent meta-analyses suggest that breastfeeding is protective against ASD^17,18^. Ghozy et al.^17^ found a reduction in the risk of ASD of 58% with ever breastfeeding, 76% with ever exclusive breastfeeding and 54% with extended breastfeeding for 6 months.

Tseng et al.^18^ also reviewed 7 articles reporting an association between breastfeeding and an ASD diagnosis. They found that children with ASD were significantly less likely to have been breastfed than those without (OR = 0.61, 95% CI = [0.45–0.83], *P* = 0.002).

However, another part of the literature qualifies the finding of an association between BF and ASD. Husk and Keim^19^ examined data from the National Survey of Children's Health from 2007 to 2011—a nationally representative survey of US children—to determine whether infant breastfeeding was associated with the later development of ASD. Approximately 38,000 respondents were asked (i) whether their child currently had ASD and (ii) to recount their child's breastfeeding history. A series of covariate-controlled analyses of the NSCH indicated that a current diagnosis of ASD (n = 391) was unassociated with any measure of BF history. The authors speculate that previous reports of such associations might reflect methodological limitations. Another study of 673 children diagnosed with ASD and 876 controls^20^ found that the initiation of BF was not associated with ASD after adjustment for child and mother socioeconomic and pregnancy characteristics. However, in this study, the mothers of children with ASD were less likely to report long duration of BF. This contrasts with results of other studies^21,22^ which found that longer duration of breastfeeding protects against autistic traits or ASD diagnoses.

There is an important heterogeneity in the clinical presentation of ASD related to intellectual abilities, symptom severity and behavioural patterns, these factors all being involved in the outcome trajectories^2,23^. To date, no study has investigated whether breastfeeding is also a protective factor for the severity of the clinical presentation of children diagnosed with ASD.

Our aim was to investigate the association between first the initiation of breastfeeding and clinical presentation severity in ASD, and second the duration of any and predominant breastfeeding and clinical presentation severity in ASD.

## Results

### Study sample

Among the 243 children included, aged 10.3 years (± 3.9), 81.9% (n = 199) were males. At inclusion of participants in the ELENA cohort, their mean ADOS-2 CSS score was 6.8 (± 2.0), their mean IQ was 77.1 (± 28.0) and their SRS-2 T-score was 94.9 (± 19.0). Their mean VABS-II scores were 72.6 (± 15.5) for communication, 75.6 (± 12.8) for daily living skills and 70.9 (± 10.6) for socialisation. Their mean ABC scores were 33.1 (± 21.0) for irritability/ aggressiveness, 26.6 (± 18.4) for lethargy/ social withdrawal, 29.6 (± 22.9) for stereotyped behaviours/ self-harm and 44.0 (± 25.0) for hyperactivity/ lack of cooperation. On average, children included in the analyses had higher IQ, VABS-II daily living skills and communication scores than in the whole ELENA cohort (see Supplementary Table S2 online).

### Birth conditions, family and socio-demographic characteristics

Of the 243 children in our sample, at the time of birth, 93% had parents (n = 226) who were living together and 53% did not have any siblings. The average age of the mothers was 31.8 years. For about 36% (n = 87), at least one of the parents had a university degree or more. Of the respondents, 72.8% (n = 177) had been breastfed. Breastfed children had parents with higher levels of education and SES (*P*  < 0.001 and *P* = 0.02, respectively) than non-breastfed children, and they were more often only children (*P* < 0.001) (Table 1).

**Table 1:** Characteristics of participants at the time of birth.

	All	Breastfed	Never-Breastfed	*P* value
	(n = 243)	(n = 177)	(n = 66)	
**Children’s characteristics**				
**Sex**				
Boy	199 (81.9%)	142 (80.2%)	57 (86.4%)	0.27
Girl	44 (18.1%)	35 (19.8%)	9 (13.6%)	
**Gestational age**				
Preterm < 37 weeks	21 (9.3%)^h^	13 (8.0%)^g^	8 (12.5%)^b^	0.29
Term ≥ 37 weeks	206 (90.7%)	150 (92.0%)	56 (87.5%)	
**Delivery mode**				
Vaginal	194 (79.8%)	137 (77.4%)	57 (86.4%)	0.12
Caesarean section	49 (20.2%)	40 (22.6%)	9 (13.6%)	
**Mother’s characteristics**				
**Age at delivery** (years)	31.77 (± 5.35)	31.44 (± 5.12)	32.67 (± 5.88)	0.13
**Obese before pregnancy** (yes)^i^	22 (9.4%)^f^	13 (7.6%)^e^	9 (14.3%)^c^	0.12
**Body Mass Index before pregnancy** (kg/m^2^)	22.6 (20.0–25.6)^f^	22.6 (20.0–25.6)^e^	22.2 (20.0–25.6)^c^	0.89
**Tobacco smoking during pregnancy** (yes)	38 (15.8%)^c^	26 (14.9%)^b^	12 (18.5%)^a^	0.5
**Socio-environmental characteristics**				
**Parental education level**^j^				
Below high-school diploma	19 (7.9%)^a^	8 (4.5%)^a^	11 (16.7%)	**< 0.001**
High school to 2-year university degree	136 (56.2%)	89 (50.6%)	47 (71.2%)	
3-year university degree	87 (35.9%)	79 (44.9%)	8 (12.1%)	
**Parental socioeconomic status (SES)**^k^				
Low SES	81 (33.6%)^b^	50 (28.4%)^a^	31 (47.7%)^a^	**0.02**
Middle SES	81 (33.6%)	65 (36.9%)	16 (24.6%)	
High SES	79 (32.8%)	61 (34.7%)	18 (27.7%)	
**Number of older siblings**				
0	127 (53.1%)^d^	101 (58.0%)^c^	26 (40.0%)^a^	**< 0.001**
1	68 (28.5%)	53 (30.5%)	15 (23.1%)	
≥ 2	44 (18.4%)	20 (11.5%)	24 (36.9%)	
**Marital status at birth**				
Single mother	17 (7.0%)	13 (7.3%)	4 (6.1%)	0.73
Parents living together	226 (93.0%)	164 (95.7%)	62 (93.9%)	
**Professional status 6 months after birth**				
Unemployed	142 (58.4%)	98 (55.4%)	44 (66.7%)	0.11
Employed or involved in training/studies	101 (41.6%)	79 (44.6%)	22 (33.3%)	

Data are presented as the mean (± SD), median (IQR) or n (%).

^a^1 missing value.

^b^2 missing values.

^c^3 missing values.

^d^4 missing values.

^e^5 missing values.

^f^8 missing values.

^g^14 missing values.

^h^16 missing values.

^i^Obese: body mass index ≥ 30 kg/m^2^.

^j^Parental education: highest level of education of one of the parents.

^k^Low SES: farm workers, labourers, service workers and unemployed; Medium SES: farmers, supervisors, skilled craftsmen; High SES: business owners, professionals, managers.

### Breastfeeding conditions

The median duration of ABF was 6 months (IQR = [2.7–9.0]), with 47.5% of children breastfed for less than 6 months (n = 84), 34.5% (n = 61) for 6–12 months and 18% (n = 32) for more than 12 months. The median duration of PBF was 3.5 months (IQR = [1.0–5.0]), with 78% of children mainly breastfed for less than 6 months (n = 138).

### Association between breastfeeding and severity of clinical presentation

Breastfed children had higher VABS-II scores for daily living skills than those who had never been breastfed (77.1 (± 12.7) vs. 71.5 (± 12.2), respectively, p = 0.002). They also tended to have higher VABS-II communication scores (73.7 (± 15.2) vs. 69.8 (± 15.9), respectively, *P* = 0.08). Breastfeeding was not associated with the VABS-II socialisation score, the SRS-2 T-score, ADOS-2 CSS, IQ or the ABC scores (Table 2).

**Table 2:** Association between breastfeeding initiation status and ASD clinical severity at inclusion.

	Breastfed	Never-Breastfed	*P* value
**Intellectual quotient**	(*N* = 177)	(*N* = 66)	
IQ	78.2 (± 28.0)	73.9 (± 28.0)	0.35
**VABS-II standard scores**	(*N* = 177)	(*N* = 66)	
Communication	73.7 (± 15.2)	69.8 (± 15.9)	0.08
Daily living skills	77.1 (± 12.7)	71.5 (± 12.2)	**0.002**
Socialisation	71.4 (± 11.2)	69.7 (± 8.8)	0.50
**ADOS-2 CSS**	(*N* = 157)	(*N* = 55)	
	6.8 (± 1.9)	6.7 (± 2.1)	0.60
**ABC**	(*N* = 136)	(*N* = 49)	
Irritability/ aggressiveness	31.1 (15.6–46.7)^a^	31.1 (17.8–46.7)	0.85
Lethargy/ social withdrawal	24.0 (12.5–37.5)	27.1 (16.7–33.3)	0.98
Stereotyped behaviours/ self-harm	23.8 (9.5–47.6)	23.8 (9.5–61.9)	0.66
Hyperactivity/ lack of cooperation	43.8 (25.0–65.6)	41.7 (14.6–58.3)	0.21
**SRS-2 T-score**	(*N* = 110)	(*N* = 38)	
	94.9 (± 18.8)	95.0 (± 19.7)	0.97

Data are presented as the mean (± SD) or median (IQR).

*VABS-II* vineland second version, *ADOS-2 CSS* autism diagnostic observation schedule second version calibrate severity scale, *ABC* Aberrant Behavior Checklist, *SRS-2* Social-Responsiveness Scale, second version.

^a^1 missing value.

Significant values are in bold.

After adjustment for potential confounders, breastfeeding was not significantly associated with VABS-II scores for daily living skills (*P* = 0.09) or communication (*P*  = 0.76) (Table 3).

**Table 3:** Adjusted multiple linear regression models to explain VABS-II Daily living skills and communication scores (n = 243).

	VABS-II Daily living skills			VABS-II Communication		
	R^2^	β (95% IC)	*P* value	R^2^	β (95% IC)	*P* value
	0.12			0.11		
***Mothers***						
**Age** (years)		0.15 (− 0.17; 0.46)	0.36		0.31 (− 0.07; 0.69)	0.11
**Tobacco smoking during pregnancy**^d^ (yes)		1.24 (− 3.21; 5.69)	0.59		0.87 (− 4.53; 6.26)	0.75
**Obesity before pregnancy**^a^^,d^ (yes)		0.79 (− 5.41; 6.99)	0.8		− 5.28 (− 12.38; 1.82)	0.15
***Child***						
**Girl**		− 3.82 (− 8.03; 0.38)	0.07		− 0.77 (− 5.87; 4.33)	0.77
**Gestational age**^d^ (ref : ≥ 37 weeks)						
< 37 weeks		− 0.16 (− 5.72 ; 5.40)	0.96		− 3.41 (− 10.17 ; 3.35)	0.32
***Social environment***						
**Parental education level**^b^^,d^ (ref: Below high-school diploma)						
High school to 2-year university degree		3.50 (− 2.97; 90.98)	0.29		6.09 (− 1.70; 13.89)	0.13
≥ 3-year university degree		6.15 (− 1.19; 13.48)	0.1		8.56 (− 0.24; 17.37)	0.06
**Number of older siblings**^d^ (ref: no sibling)						
*1*		− 3.85 (− 7.58; − 0.12)	0.04		− 4.68 (− 9.22; − 0.15)	0.04
≥ *2*		− 5.10 (− 9.86; − 0.35)	0.04		− 7.00 (− 12.80; − 1.21)	0.02
**Parental socioeconomic status (SES)**^c^^,d^ (ref: High SES)						
Low SES		− 2.84 (− 7.25; 1.58)	0.21		− 1.07 (− 6.42; 4.28)	0.7
Middle SES		0.14 (− 3.83; 4.11)	0.94		− 0.95 (− 5.77; 3.86)	0.7
**Ever breastfed** (yes)		3.37 (− 0.47; 7.20)	0.09		0.71 (− 3.94; 5.36)	0.76

^a^Obese: body mass index ≥ 30 kg/m^2^.

^b^Parental education: highest level of education of one of the parents.

^c^Low SES: farm workers, labourers, service workers and unemployed; Medium SES: farmers, supervisors, skilled craftsmen; High SES: business owners, professionals, managers.

^d^Multiple imputations.

The 31 children (12.8%) with incomplete data had a lower VABS-II daily living score than those with complete data (71.6 (± 11.0) vs. 76.2 (± 13.0), respectively, *P* = 0.04). Analysis of complete records showed a significant association between breastfeeding and the VABS-II score for daily living skills (adjusted β [95% CI]: 5.15 (1.13; 9.17), *P* = 0.01) but confirmed the absence of association between breastfeeding and the VABS-II communication score (*P*  = 0.35).

### Association between duration of breastfeeding and severity of clinical presentation

In breastfed children, neither the duration of ABF nor the duration of PBF were significantly associated with the severity of clinical presentation for any of the variables considered (VABS-II, SRS-2 T-score, ADOS-2 CSS, IQ and ABC scores; all Spearman's rho < 0.10, with non-significant p-value). Furthermore, no dose effect of ABF or PBF was found on the severity of clinical presentation (see Supplementary Table S3 online).

## Discussion

This study examined the relationship of (i) the initiation of breastfeeding and (ii) its duration with the severity of clinical presentation of children diagnosed with ASD according to DSM5 criteria and a multidimensional assessment.

In our sample of 243 children with a confirmed diagnosis of ASD, 73% had been breastfed, which is comparable to the general French population^24–26^. This contradicts a meta-analysis that found that children with ASD were breastfed less often than the general population suggesting a protective role of breastfeeding against ASD^18^. However, this result was not confirmed when the diagnosis of ASD had to be made strictly according to DSM criteria (as in our study) and not by parental self-reports. Furthermore, this result was not replicated in a study published by Soke et al.^20^ (not included in Tseng's meta-analysis) when adjustment was made for confounding factors, as also done in our study.

We did not find any gender difference in our study regarding the prevalence of breastfeeding among children with ASD, which is also in line with the observation in the general population^12^. We found a 52% rate of children with ASD still being breastfed at six months of age. This is higher than the 19% rate reported in the general paediatric population^25^, but consistent with the 51% rate reported in another population of children with ASD by Soke et al.^20^.

In our study, children with ASD who were breastfed had better daily living skills and tended to have better communication skills, according to VABS-II scores, than those who were not. This intergroup difference was not significant after adjusting for a large set of confounding factors related to the child, mother and social environment characteristics. However, our sensitivity analysis carried out only on the children for whom we had all the adjustment variables showed that the initiation of breastfeeding was significantly associated with daily living skills. We assume that this discrepancy was related to a selection bias, the 31 children with missing adjustment variables and who were not included in the sensitivity analysis, having significantly lower daily living skills than those without missing variables.

We did not find any association between the initiation of breastfeeding and the severity of ASD symptoms measured by the ADOS-2 CSS or the SRS-2, which cannot be compared with the literature in the absence of published data. Furthermore, in contrast with the general population^9,27,28^, our results do not support a protective effect of breastfeeding on the IQ of children with ASD.

Although there is some evidence in previous literature that longer duration of any and predominant breastfeeding are significantly associated with a decreased risk of ASD^22^, our results do not support a protective role against greater clinical severity in ASD. The evidence of a positive association between breastfeeding duration and IQ in the general population^9,10,12,29^ was not found in our sample of children with ASD.

This study is one of the first to examine the association between breastfeeding and the severity of clinical presentation in children with ASD. One of the strengths of our study is the large sample of children with a confirmed diagnosis of ASD following a multidisciplinary clinical assessment and using DSM-5 criteria. In addition, the assessment of clinical presentation was performed using standardised tools that target multiple dimensions, such as ASD symptoms, IQ, adaptive behaviours and behavioural problems^30^. We also considered a wide range of potential confounders, including parental socioeconomic and educational levels^16^.

Our study has several limitations that need to be taken into account when interpreting the results. First, data on breastfeeding were collected retrospectively when the children were, on average, 10 years old (± 3.86), which implies a risk of recall bias. However, it has been suggested that mothers' recall of breastfeeding initiation and duration are accurate, even 6 to 20 years after childbirth^31–34^. Second, because participants were recruited from tertiary centres, such as autism resource centres, the study population may not be fully representative of the general population with ASD. Third, the children included in our study differed from the other participants in the ELENA cohort in that they had higher levels of IQ and adaptive skills, which limits the generalisability of our results. Maternal IQ^16,35^ and autistic traits^20^, which are also known to be potential confounders, were not taken into account in our study. Finally, regarding the protective effect of breastfeeding, the population size of our study is limited compared to other studies, some involving several thousand participants. It is therefore not possible to exclude a possible association between breastfeeding and severity of clinical presentation in ASD. However, given the limitations of our study and of those published on breastfeeding in ASD, prospective studies of populations at risk of ASD are necessary. These need to be based particularly on large prenatal cohorts, making it possible to take confounding factors into account, including family socio-demographic factors and maternal metabolic conditions, by means of a very large data collection.

## Methods

This retrospective survey, carried out between September 20 and November 22, 2021, was nested in the ELENA cohort^36^, a prospective, multiregional and observational study of children with a confirmed diagnosis of ASD followed for a period of six years. Signed informed consent was obtained from a parent and/or legal guardian for the study participation. The study design and protocol have previously been thoroughly described^36^. This study was approved by the Review Board of the University Hospital of Montpellier, France. All methods were performed in accordance with the relevant guidelines and regulations.

### Participants

Among the 876 children with a confirmed diagnosis of ASD included in the ELENA cohort, 243 (27.7%) parents had fully completed the breastfeeding section in the *Early Feeding Patterns Questionnaire*. The mean age of the 243 participants was 10.3 years (± 3.9) and the age range was 4 to 22 years. The sample characteristics are detailed in Table 1.

### Measurements

#### Data collection

Parents were sent an email inviting them to participate in the study by completing an online questionnaire on early feeding patterns. Parents who did not complete the questionnaire were contacted again two weeks and one month after the initial request, by email and phone. Only one questionnaire was to be completed per child and by at least one parent. The questionnaire was either self-administered online, administered by phone with the help of a clinical research assistant or completed using a printed version mailed back to our research team.

#### Early feeding patterns questionnaire

Detailed information was obtained using a parental self-questionnaire adapted from the EDEN cohort^37^. It focused on (i) parents at the time of the birth: age, education level, socio-economic status (SES), occupation, household composition, mother's weight, height and body mass index before pregnancy, maternal smoking; (ii) birth of the child: gestational age, delivery, birth weight; and (iii) feeding patterns of the newborn in the first year of life: initiation and duration of breastfeeding, respective ages at first formula feeding and solid foods.

#### Early feeding patterns

Mothers who initiated breastfeeding were identified by the following question: "Did you breastfeed your baby, even for a few hours?”. The duration in months of any breastfeeding (ABF, including partial or exclusive breastfeeding) was then determined by the question: "How old was your baby when you stopped breastfeeding?”. The duration in months of predominant breastfeeding (PBF—where the infant also receives water or other liquids^38^) was determined by asking the age of initiation of solid foods and/or formula. To facilitate their answer, parents were asked to use the child's health record where this information on breastfeeding and early feeding is usually found.

#### Child assessment

Severity of the clinical presentation was determined through the assessment of autism symptoms, intellectual level, adaptive behaviours and behavioural problems. These data were collected at the time of inclusion in the ELENA cohort, when the children were on average 5.8 years old (standard deviation (SD) = 3.4). The severity of autism symptoms was measured using the Autism Diagnostic Observation Schedule-2 (ADOS-2) total algorithm score (calibrated severity score, CSS)^39,40^. The ADOS-2 is a standardised assessment tool based on a set of age-appropriate structured or semi-structured tasks. A total score of 5–7 suggests moderate related symptoms and > 8 high related symptoms. The intellectual quotient (IQ) was calculated using standardised and validated instruments (Brunet-Lézine-R^41^, BECS^42^, PEP-3^43^, WPPSI-IV^44^, WISC-V^45^, WAIS-IV^46^, K-ABC^47^) selected according to the child's age and developmental level following the approach used by Howlin et al.^48^. Adaptive skills were assessed using the Vineland Adaptive Behavior Scales, Second Edition (VABS-II), a standardised, semi-structured parent interview^49^. Standard scores (mean = 100, SD = 15) were obtained for the three sub-domains: communication, daily living skills, and socialisation. Deficits in social behaviour were assessed using the Social-Responsiveness Scale, second version (SRS-2), administered to parents^50,51^. Gender-standardised total T-scores were used. Behavioural problems were measured with the Aberrant Behavior Checklist (ABC) completed by parents^52^.

### Statistical analysis

The main exposures were the initiation of breastfeeding and the durations of ABF and PBF. The outcome was the child’s clinical presentation severity defined by a set of variables (ADOS-2 CSS, VABS-II standard scores in communication, socialisation and daily living skills, IQ, SRS-2 T-total score and ABC scores for irritability/aggressiveness, lethargy/social withdrawal, stereotyped behaviours/self-harm and hyperactivity/lack of cooperation). In our analysis, we took into account multiple confounding factors considered in previous studies on the association between breastfeeding and children's cognitive development^16^: child's sex, gestational age, mother's age at delivery, maternal obesity or smoking during pregnancy, education and socio-economic status of the household and the presence of siblings.

Continuous variables are described by means ± SDs or medians with interquartile ranges (IQRs) and categorical variables by frequencies and percentages. The Chi-square or Fisher's test and the Wilcoxon Mann–Whitney test or ANOVA were used, as appropriate, to compare differences between children: breastfed or never, included in the study or not, with or without missing data for confounders.

We evaluated associations of (i) the initiation of breastfeeding and (ii) the durations of ABF and PBF among breastfed children on clinical severity outcomes using ANOVA and Spearman’s correlations respectively. When the p-value of the effect was < 0.20, a multiple linear regression analysis adjusted for the confounding variables listed above was computed. The chained equations missing data method^53^ was used to impute the missing confounder values (15 imputation datasets), assuming that the data were randomly missing. The missing data description is presented in Supplementary Table S1. The goodness of fit was assessed by the R^2^. A sensitivity analysis was performed to investigate the effect of missing data using a complete case analysis. Among breastfed children, a possible dose–effect of the duration of ABF or PBF on clinical severity outcomes was assessed using ANOVA. ABF and PBF were used in tertiles: low (< 2 months), middle (2–4.5 months) and high (≥ 4.5 months) for PBF duration and low (< 3 months), middle (3–8 months) and high (≥ 8 months) for ABF duration. All statistical tests were considered significant for *P*  < 0.05. Analyses were performed using SAS Enterprise Guide V7.13 (SAS Institute Inc., Cary, NC, USA).

### Ethical approval

The study and informed consent procedure have been approved by the Internal Review Board of the University Hospital of Montpellier. The ELENA cohort has been approved by the Ethics Committee on the Research of Human Subjects at Marseille Mediterranean and the National Commission for Computing and Liberties (CNIL number DR-2015–393).

### Consent to participate

Signed informed consent has been obtained from all participating families included in the ELENA cohort.

## Supplementary Information

Supplementary Information 1.

Supplementary Information 2.

## Data Availability

The datasets used and analysed during the current study are available from the corresponding author upon reasonable request.
