# Supplementary material for: Breastfeeding is not a risk factor for clinical severity in Autism spectrum disorder in children from the ELENA cohort
Source: Sci Rep. 2023 Jan 16;13:816. doi: 10.1038/s41598-022-27040-x (PMC9842713; doi:10.1038/s41598-022-27040-x)
Supplement: Supplementary file 1 — Supplementary Information 1. [file 41598_2022_27040_MOESM1_ESM.pdf]

Breastfeeding is not a risk factor for clinical severity in Autism Spectrum Disorder in children from the ELENA cohort

Scientific Report

Marianne Peries[1,2,3], Fanny Duhr[1], Marie-Christine Picot[4,3,2], Barbara Heude[5], Jonathan Y. Bernard[5,6], Amaria Baghdadli[1,2,3,7]

[1] Centre de Ressource Autisme Languedoc-Roussillon, CHU Montpellier, Montpellier, France

[2] Centre d'Excellence sur l'Autisme et les Troubles Neuro-développementaux, CHU Montpellier, France

[3] Université Paris-Saclay, UVSQ, Inserm, CESP, Team DevPsy, 94807, Villejuif, France

[4] Clinical research and epidemiology unit, Department of Medical Information, University Hospital, CHU Montpellier, France

[5] Université Paris Cité, Inserm, INRAE, Centre de Recherche en Épidémiologie et Statistiques, F-7005 Paris, France

[6] Singapore Institute for Clinical Sciences (SICS), Agency for Science, Technology and Research (A*STAR), Singapore, Singapore

[7] Faculté de Médecine, Université de Montpellier, France

Corresponding author: Amaria BAGHDADLI, Centre de Ressource Autisme Languedoc-Roussillon et Centre d'Excellence sur l'Autisme et les Troubles Neuro-développementaux (CeAND), 39 Avenue Charles Flahaut, 34295 Montpellier cedex 05, France. Tel: +33467330986 ; Fax: +33467330832 ; E-mail address: rech-clinique-autisme@chu-montpellier.fr

Supplementary Table S1. Description of missing data for each confounders included in the multiple imputation models

Confounders	Missing value n (%)
Mothers' age (years)	0
Mother's tobacco smoking during pregnancy	3 (1.2)
Mother's obesity before pregnancy	8 (3.3)
Child's sex	0
Gestational age (weeks)	16 (6.6)
Parental education level	1 (0.41)
Number of older siblings	4 (1.64)
Parental socioeconomic status (SES)	2 (0.8)

Supplementary Table S2. Comparison of clinical characteristics at inclusion between participants considered in the analyses and the other participants involved in the ELENA Cohort

	**Participants included in our study (n = 243)**	**Other participants included in ELENA Cohort (n = 633)**	**p value**
<u>**Children characteristics at assessment**</u>			
**Age** (years)	5.8 (±3.4)	6.0 (±3.3)	0.34
**Sex**			
Boy	199 (81.9%)	525 (82.9%)	0.72
Girl	44 (18.1%)	108 (17.1%)	
**Intellectual quotient**	**77.1 (±28.0)**	**71.3 (±25.7)**	**0.01**
**VABS-II standard scores**			
Communication SS	**72.6 (±15.5)**	**68.9 (±15.2)[a]**	**0.001**
Daily living skills SS	**75.6 (±12.8)**	**72.5 (±12.7)**	**0.001**
Social skills SS	70.9 (±10.6)	69.5 (±10.8)[a]	0.20
**Level of autism spectrum (ADOS-2 CSS)**			
No evidence to low related symptoms	29 (13.7%)[b]	61 (10.8%)[c]	0.47
Moderate related symptoms	100 (47.1%)	263 (46.6%)	
High related symptoms	83 (39.2%)	240 (42.6%)	
**ABC**			
Irritability / aggressiveness	31.1 (17.8 - 46.7)[d]	31.1 (17.8 - 47.8)[g]	0.55
Lethargy / social withdrawal	25.0 (12.5 - 35.4)[e]	25.0 (14.6 - 37.5)[h]	0.56
Stereotyped behaviors / self-harm	23.8 (9.5 - 47.6)[e]	28.6 (14.3 - 52.4)[h]	0.10
Hyperactivity / lack of cooperation	43.8 (20.8 - 64.6)[e]	43.6 (27.1 - 60.4)[i]	0.88
**SRS-2 T-score**	94.9 (±19.0)[f]	93.9 (±18.3)[j]	0.62
<u>**Parents characteristics**</u>			
**Age at birth** (years)			
Mother	31.8 (±5.4)	31.2 (±5.4)	0.24
Father	35.3 (±7.3)	34.2 (±6.4)	0.16
**Mother's education level**			
High school or lower	66 (34.4%)	145 (41.4%)	0.11
University	126 (65.5%)	205 (58.6%)	
**Father's education level**			
High school or lower	91 (47.6%)	168 (49.1%)	0.74
University	100 (52.4%)	174 (50.9%)	

Data are presented as the mean (±SD), median (IQR), or n (%).
[a] 1 missing value, [b] 31 missing values, [c] 69 missing values, [d] 59 missing values, [e] 58 missing values, [f] 95 missing values, [g] 321 missing values, [h] 323 missing values, [i] 320 missing values, [j] 410 missing values
ADOS-2 CSS, autism diagnostic observation schedule second version calibrate severity scale; VABS-II, Vineland second version; ABC, Aberrant Behavior Checklist; SRS-2, Social-Responsiveness Scale, second version.

Supplementary Table S3. Association between predominant and any breastfeeding in tertiles and ASD clinical severity at inclusion

	**Predominant Breastfeeding**				**Any Breastfeeding**			
	**Low tertile** (<2 months)	**Middle tertile** (2-4.5 months)	**High tertile** (≥4.5 months)	**P value**	**Low tertile** (<3 months)	**Middle tertile** (3-8 months)	**High tertile** (≥8months)	**P value**
**Intellectual quotient**	*(N = 60)*	*(N = 76)*	*(N = 41)*		*(N = 62)*	*(N = 61)*	*(N = 54)*	
IQ	74.0 (±24.4)	82.2 (±30.7)	77.2 (±27.1)	0.23	77.5 (±24.4)	76.2 (±30.0)	81.4 (±29.6)	0.60
**VABS-II standard scores**	*(N = 60)*	*(N = 76)*	*(N = 41)*		*(N = 62)*	*(N = 61)*	*(N = 54)*	
Communication	73.8 (±14.0)	74.8 (±14.3)	71.5 (±18.4)	0.53	74.5 (±14.1)	71.8 (±14.9)	74.9 (±16.8)	0.47
Daily living skills	77.9 (±11.7)	76.9 (±13.0)	76.4 (±13.9)	0.82	76.9 (±11.8)	77.4 (±13.2)	77.1 (±13.5)	0.98
Socialization	73.0 (±10.4)	70.1 (±11.3)	71.4 (±12.1)	0.33	71.3 (±11.3)	71.23 (±11.4)	71.54 (±11.1)	0.99
**ADOS-2 CSS**	*(N = 52)*	*(N = 70)*	*(N = 35)*		*(N = 53)*	*(N = 56)*	*(N = 48)*	
	7.0 (±1.5)	6.6 (±2.3)	6.9 (±1.8)	0.75	6.8 (±1.9)	7.2 (±1.9)	6.4 (±2.0)	0.09
**ABC**	*(N = 43)*	*(N = 63)*	*(N = 30)*		*(N = 47)*	*(N = 48)*	*(N = 41)*	
Irritability/ aggressiveness	26.7 (11.1 - 40.0)	33.3 (20.0 - 51.1)[a]	37.8 (20.0 - 44.4)	0.14	26.7 (13.3 - 46.7)	31.1 (13.3 - 42.2)[a]	37.8 (20.0 - 48.9)	0.30
Lethargy/ social withdrawal	22.9 (12.5 - 35.4)	22.9 (10.4 - 41.7)	27.1 (12.5 - 35.4)	0.85	22.9 (12.5 - 43.8)	22.9 (10.4 - 38.5)	25.0 (12.5 - 31.3)	0.91
Stereotyped behaviors/ self-harm	19.1 (4.8 - 42.9)	28.6 (9.5 - 47.6)	23.8 (9.5 - 52.4)	0.28	28.6 (9.5 - 47.6)	26.2 (9.5 - 47.6)	23.8 (9.5 - 42.9)	0.99
Hyperactivity/ lack of cooperation	35.4 (20.8 - 62.5)	45.8 (20.8 - 68.8)	50.0 (37.5 - 64.6)	0.23	37.5 (22.9 - 64.6)	43.8 (22.9 - 65.6)	50.0 (31.3 - 66.7)	0.63
**SRS-2 T-score**	*(N = 36)*	*(N = 51)*	*(N = 23)*		*(N = 40)*	*(N = 39)*	*(N = 31)*	
	93.3 (±18.1)	93.8 (±17.9)	99.7 (±21.7)	0.38	93.3 (±18.6)	98.7 (±18.7)	91.9 (±19.1)	0.26

Data are presented as the mean (±SD) or median (IQR).
[a] 1 missing value
VABS-II, Vineland second version; ADOS-2 CSS, autism diagnostic observation schedule second version calibrate severity scale; ABC, Aberrant Behavior Checklist; SRS-2, Social-Responsiveness Scale, second version.
