# Supplementary material for: Breastfeeding is not a risk factor for clinical severity in Autism spectrum disorder in children from the ELENA cohort
Source: Sci Rep. 2023 Jan 16;13:816. doi: 10.1038/s41598-022-27040-x (PMC9842713; doi:10.1038/s41598-022-27040-x)
Supplement: Supplementary file 2 — Supplementary Information 2. [file 41598_2022_27040_MOESM2_ESM.docx]

Supplementary Information: ELENA Study group members

| **ELENA Study group members** | **Affiliations** |
| --- | --- |
| Prof. Amaria Baghdadli | Centre de Ressource Autisme Languedoc-Roussillon et Centre d’Excellence sur l’Autisme et les Troubles Neuro-développementaux (CeAND), CHU Montpellier, Montpellier, France  Université Paris-Saclay, UVSQ, Inserm, CESP, Team DevPsy, 94807, Villejuif, France  Faculté de Médecine, Université de Montpellier, France |
| Dr. Catherine Chabaux | Centre de Ressources Autisme Alsace, CHRU Strasbourg, Strasbourg, France |
| Dr. Clarisse Chatel | Centre de Ressources Autisme PACA antenne de Marseille, Service de Psychiatrie Infanto-Juvénile, Hôpital Sainte-Marguerite, Marseille, France |
| Prof. David Cohen | Service de psychiatrie de l'enfant et de l'adolescent, Groupe Hospitalier de la Pitié Salpetrière, Paris, France |
| Dr. Emmanuel Damville | Centre de Ressources Autisme Nord-Pas de Calais, CHRU Lille, Lille, France |
| Dr. Marie-Maude Geoffray | Centre d’Evaluation et de Diagnostic de l’Autisme (CEDA) et autres Troubles du Neurodéveloppement, CH le Vinatier, Bron, France |
| Prof. Ludovic Gicquel | Centre de Ressources Autisme Poitou-Charentes, CH Henri Laborit, Poitiers, France |
| Prof. Renaud Jardri | Centre de Ressources Autisme Nord-Pas de Calais, CHRU Lille, Lille, France |
| Dr. Thierry Maffre | Centre de Ressources Autisme Midi-Pyrénées, Hôpital La Grave, Toulouse, France |
| Dr. Alexandre Novo | Centre de Ressource Autisme Champagne-Ardenne, CHU de Reims, Reims, France |
| Dr. Roxane Odoyer | Centre de Ressources Autisme Midi-Pyrénées, Hôpital La Grave, Toulouse, France |
| Dr. Marie-Joëlle Oreve | CH Versailles, Versailles, France |
| Dr. Didier Périsse | Service de psychiatrie de l'enfant et de l'adolescent, Groupe Hospitalier de la Pitié Salpetrière, Paris, France |
| Prof. François Poinso | Centre de Ressources Autisme PACA Marseille, Service de Psychiatrie Infanto-Juvénile, Hôpital Sainte-Marguerite, Marseille, France |
| Dr. Julien Pottelette | Centre de Ressources Autisme Alsace, CHRU Strasbourg, Strasbourg, France |
| Dr. Laurence Robel | Service de psychiatrie de l'enfant et de l'adolescent, Hôpital Necker, Paris, France |
| Prof. Catherine Rolland | Centre de Ressource Autisme Champagne-Ardenne, CHU de Reims, Reims, France |
| Dr. Marie Schoenberger | Centre de Ressources Autisme Lorraine, Centre Psychothérapique de Nancy, Laxou, France |
| Dr. Sandrine Sonié | Centre de Ressources Autisme Rhône-Alpes, CH le Vinatier, BRON, France |
| Prof. Mario Speranza | CH Versailles, Versailles, France |
| Dr. Stéphanie Vespérini | Centre de Ressources Autisme PACA antenne de Nice, Service Universitaire de psychiatrie de l’Enfant et de l’Adolescent du Professeur F. ASKENAZY, CHU Lenval, Nice, France |
